# An IoT Architecture for Water Resource Management in Agroindustrial Environments: A Case Study in Almería (Spain)†

**DOI:** 10.3390/s20030596

**Published:** 2020-01-21

**Authors:** Manuel Muñoz, Juan D. Gil, Lidia Roca, Francisco Rodríguez, Manuel Berenguel

**Affiliations:** 1 Departamento de Informática, Escuela Superior de Ingeniería, Universidad de Almería, ceiA3, CIESOL, Ctra. Sacramento s/n, 04120 Almería, Spain; juandiego.gil@ual.es (J.D.G.); frrodrig@ual.es (F.R.); beren@ual.es (M.B.); 2 Convenio Universidad de Almería, Plataforma Solar de Almería, Ctra. Senés s/n, 04200 Tabernas, Almería, Spain; lidia.roca@psa.es

**Keywords:** FIWARE, cloud storage, model predictive control, smart water management, smart agriculture

## Abstract

The current agricultural water panorama in many Mediterranean countries is composed by desalination facilities, wells (frequently overexploited), the water public utility network, and several consumer agents with different water needs. This distributed water network requires centralized management methods for its proper use, which are difficult to implement as the different agents are usually geographically separated. In this sense, the use of enabling technologies such as the Internet of Things can be essential to the proper operation of these agroindustrial systems. In this paper, an Internet of Things cloud architecture based on the FIWARE standard is proposed for interconnecting the several agents that make up the agroindustrial system. In addition, this architecture includes an efficient management method based on a model predictive control technique, which is aimed at minimizing operating costs. A case study inspired by three real facilities located in Almería (southeast of Spain) is used as the simulation test bed. The obtained results show how around 75% of the total operating costs can be saved with the application of the proposed approach, which could be very significant to decrease the costs of desalinated water and, therefore, to maintain the sustainability of the agricultural system.

## 1. Introduction

Almería (southeast of Spain) is one of the driest regions in Europe, but paradoxically, it has one of the continent’s largest agricultural production systems. Such a system is composed of more than 30,000 ha of effective greenhouse production [1], and it has become the main driving force of the economy of this dry region. One basic ingredient of this system is fresh water, so that the development of agriculture in Almería has been associated for many years with the decline of fresh water reservoirs, despite being the agricultural area where the most efficient management of this resource is carried out [2]. This encouraged the installation of desalination plants as a tool to maintain the fresh water availability in the region and, therefore, the sustainability of the agricultural system [3]. Thus, Almería’s current agricultural water panorama comprises consumer agents, as greenhouses and industries related to agriculture, and producer agents based on conventional (water public utility network and wells) and non-conventional sources (desalination facilities). This agroindustrial environment constitutes a distributed water network that requires an integral smart management method for its optimal use [4].

Several management strategies have been formulated in the literature for distributed water networks, especially focused on the urban water cycle of Barcelona (Spain). In the work of Ocampo Martinez et al. [5], Model Predictive Control (MPC) paradigms were proposed aimed at reducing pumping costs. An operational MPC approach was proposed by Pascual et al. [6], tasked with reducing costs, as well as maintaining safety storage volumes in the buffer tanks. Another control based water management system was proposed by Lopez Farias et al. [7]. In this case, the forecasting accuracy of the control system was improved by means of a qualitative multi-model predictor. Although all these works proposed effective management methods, they did not describe the way in which all the information coming from the different devices of the distributed network is integrated and unified for the application of the methods in real cases. In addition, they are focused on optimizing the transport water network, without taking into account the optimal management of the water sources.

To manage the water sources optimally, it is essential to adapt the production to the demand. This fact is especially significant when considering desalination facilities in the water network (as happens in Almería) as the costs associated with the production of desalinated water are still relatively high [8], and they depend directly on production. The work presented by Roca et al. [9] demonstrated how a desalination facility can be efficiently coupled to a greenhouse by using MPC techniques. Moreover, the work in [10] showed how metrics related to the desalination process can be improved in these kinds of combinations by using advanced control strategies. However, these works were focused only on the management method, considering desalination as the only water source, and without taking into account costs in the management problem. As stated in [11], to minimize the operational costs of desalination facilities and to improve their efficiency, a bi-directional communication between them and the consumers must be established, which can be achieved by means of adequate Information and Communication Technologies (ICT) tools. This is especially relevant in Almería, where there are relatively small and geographically dispersed greenhouse cultivation areas that depend on the water coming from one or several desalination plants. These plants need to know the water demand of each greenhouse for their efficient use in terms of operating costs. One natural choice to solve the problem is the Internet of Things (IoT) framework, as it allows connecting all the devices in a unified platform regardless of their geographical location. In addition, IoT can be associated with data analytics and cloud computing, thus making powerful platforms that allow gathering the required information from different IoT devices, analyzing it in the cloud, and transmitting the corresponding control signals to the actuators of the distributed network. These features have made IoT a key component in the development of sustainable distributed environments [12,13].

In recent years, IoT has experienced a breakthrough, changing the way in which providers and consumers interact with each other. Agriculture is not alien to this transition, and it is experiencing a change of the business model in the technological field [14,15] according to which customers (farmers) are ceasing to acquire assets (monitoring computers, sensors, or control systems) while demanding services [16,17]. In this framework, the supplier companies are responsible for ensuring the proper functioning of their equipment and providing the data necessary for farmers to make their decisions, billing for such services. The devices, through the change of firmware, can dynamically change the activities they carry out, supplying information to the cloud (not only locally as is usually done) [18] and, through the adequate processing of this information, help to reduce service times and operation shutdowns. This paradigm shift requires the establishment of transversal protocols, interoperability, and collaboration between companies and services.

Nevertheless, the arrival of IoT to the agricultural field not only has influenced the relationships between costumers and companies, but also the way in which the agricultural activities are developed. Thus, the term Precision Agriculture (PA) has emerged [19,20], which involves the use of a series of sensors and actuators that allow gathering context information of the environment that surrounds them. This paradigm enables the development of tools to assist in decision-making, monitoring activities in the crop, and applications to improve the quality; all of them aimed at obtaining a more efficient and sustainable agricultural system [21,22,23,24]. In this way, different agronomic data management platforms using IoT technologies are being developed, often driven by European Union initiatives. These platforms are a natural evolution of the so-called farm management systems to make them compatible with cloud computing [19,21,25]. FIWARE is an initiative of the European Union for the creation of a platform that helps the development of applications and solutions focused on the IoT [26]. FIWARE aims to create an open and sustainable ecosystem, based on public software standards.

Moreover, regarding water management in crops, which is the scope of this work, several approaches have been proposed in literature making a proper use of IoT technology for developing smart irrigation systems, some of them based on the use of FIWARE as well. In [27], an IoT system for smart energy consumption and irrigation was presented. The system decides the amount of water required by the crops according to the current moisture in soil and humidity (measured by mean of IoT sensors) and the time of the day. A similar approach was proposed in [28], but in this case, meteorological data were also considered to predict the water requirements based on a machine learning system. The most complete approach was the one proposed in [29], in which an IoT platform based on FIWARE was proposed for smart water management in agriculture taking into account water reserve, water distribution, and water consumption. However, all these works were mainly focused on the management of irrigation and on the distribution system considering only the public utility water network as the water source.

In this way, the main gaps observed in the literature according to the above review are the following ones:

- The management methods presented for distributed water networks so far only addressed the optimization of the transport water network, without taking into account water sources. Besides, they did not establish the way in which the different agents of the distributed network were interconnected.
- The works addressing the connection of non-conventional water sources as desalination facilities and consumer agents were focused on the optimal management of the desalination facilities, without considering other water sources in the problem. In addition, they were aimed at improving metrics related to the desalination process, but not to minimizing economic costs, which is very relevant for the correct implementation of this type of framework. Furthermore, they did not describe the way in which the agents are interconnected.
- The works related to enabling technologies such as IoT in the agricultural field were focused on improving the crops’ performance, addressing aspects like the optimization of the irrigation system, or the development of platforms to improve the decision making. However, to the authors’ knowledge, there are no works that discuss the use of these kinds of technologies for the effective management of agroindustrial districts in terms of water, which can be essential to optimize the performance of the new panorama that arises with the introduction of new water sources in the agricultural ecosystem.

To address the aforementioned issues, this work presents an IoT platform for the optimal management of the distributed water network of agroindustrial environments. The contributions of the work are three fold. First, a scalable IoT platform for the interconnection of the different agents composing the distributed water network is proposed. The IoT platform is based on FIWARE, and the architecture is fragmented into layers or services. Second, the IoT platform incorporates an MPC strategy tasked with optimizing the operational costs of the water network, taking into account the costs of the feed pump of the desalination facility and the costs of the water coming from the public utility network, while ensuring the water needs. Third, to demonstrate the effectiveness of the proposed approach, a case study based on three real plants located in Almería is used to carry out exhaustive simulation tests with non-optimal management methods.

The reminder of the document is organized as follows: Section 2 is dedicated to depicting the concept and problems of agroindustrial districts. Section 3 is aimed at presenting the basis of the proposed IoT platform, a general overview of it, and the description of the practical case study adopted. Section 4 presents and discusses the simulation results. Section 5 summarizes the conclusions obtained from the results and possible future work.

## 2. Agroindustrial District: Definition and Problems

Before presenting the IoT architecture, it is essential to define the agroindustrial district concept and the problems associated with this kind of system. The contemporary industrial district theory is owed to Giacomo Beccattini [30], who defined it as a territorial-partner entity characterized by the active presence of a community of people and a population of companies in a given geographical and historical space. Following this proposal, the term agroindustrial district was introduced as a district constituted by farms, processing companies, and supply companies located in a given territory. This environment is usually dominated by small and medium enterprises, specialized in one of the phases of the production chain. Among them, there are important relationships of the vertical type (between companies of different phases of the production process) and of the horizontal type (between companies of the same phase) or transversal (with supply and service companies).

In this synergy framework, there are systems with different objectives that depend on the needs of heterogeneous resources, both energy (electricity and heat/cold) and others (such as water and CO${}_{2}$). In addition, if renewable energy is included in this environment, it is necessary to manage conveniently the efficient use of all resources in each of the systems, as well as coordinate the flow between them. The research project Control and Optimal Management of Heterogeneous Resources in Agroindustrial production districts integrating renewable Energies (CHROMAE) (www2.ual.es/chromae), funded by the Spanish Ministry of Economy, Industry and Competitiveness and ERDF funds, is aimed at developing comprehensive, coordinated, and optimal management strategies for the heterogeneous resources required by the elements that make up an agroindustrial district. The different agents, resources, and interlinks considered in the CHROMAE project can by found at (www2.ual.es/chromae).

Although the present work is based on this project, it particularizes only the production phase of the agroindustrial sector, the most extended case in Almería. In this phase, the challenge was directly related to the optimal and efficient management of water, essential for crops. This resource must be managed, establishing as a main premise that the result of such optimal management produces an as small as possible environmental impact. Moreover, economic criteria must also be taken into account in the management problem. Another fact to consider and that should be added to the problem is that the different elements of the district are usually geographically separated. This requires the use of enabling technologies (such as IoT) to close the circle, interconnect different systems, and to manage all of them centrally.

## 3. The IoT Platform: Basis, Overview and Case Study Description

This section presents the main components of the IoT architecture developed for the optimal management of an agroindustrial district, as well as a general description of it and its application in a case study in Almería. In this way, the FIWARE platform and the MPC technique are firstly depicted. Then, an overview of the proposed platform is shown and described, and finally, the case study in Almería is developed.

### 3.1. FIWARE

FIWARE [26] is an IoT platform driven by the European Union. FIWARE presents a modular architecture based on open source components trying to form an open and sustainable ecosystem with the capacity to be adapted to different environments. This platform provides cloud capabilities based on OpenStack [31] including a series of libraries and tools known as Generic Enablers (GEs) [32], which facilitate the creation of applications and services in IoT. These GEs offer Representational State Transfer (REST) and an Application Programming Interface (API) with public and free specifications, which allow the integration of third party software services, leading to an acceleration in the development of intelligent solutions, including data analysis and processing, persistence, and language interpreters, among others. The GEs are based on APIs that implement the Open Mobile Alliance (OMA) and Next-Generation Services Interface (NGSI) standards [33].

FIWARE is trying to promote a new standard for IoT. The main core of FIWARE, and mandatory for its use, is the enabler called the Orion Context Broker (OCB), which is responsible for managing context information. In this sense, context refers to the entire environment surrounding the IoT system, which is capable of producing relevant information for the development of the system. This context information is generated by different data sources such as a network of sensors, existing third party applications, actuators, and other devices. The following points describe some of the most important GEs of FIWARE [32]:

1. OCB: It is the intermediary between producers (publishers) and consumers (subscribers). The NGSI interface is a RESTful APIs service, which allows queries on the status of context information. This allows creating as many entities as sensors or set of sensors available in the system, allowing collecting information in real time.
2. Cygnus: It is the GE in charge of persisting context information, and it is based on Apache Flume. Cygnus allows making a copy of the data simultaneously in different databases that include: MySQL, MongoDB, PostgreSQL, or Big Data platforms such as Hadoop or Spark. In this way, subscriptions are made to OCB entities, which notify the system of a change to store the data.
3. Intelligence Data Advanced Solution (IDAS):This GE acts as a language interpreter between the different communication protocols used in IoT to the NGSI standard. The communication protocols available are LightweightM2M (LWM2M) over Constrained Application Protocol (CoaP), JavaScript Object Notation (JSON), or UltraLight over Hypertext Transfer Protocol (HTTP)/Message Queue Telemetry Transport protocol (MQTT), or Object Linking and Embedding for Process Control-Unified Architecture (OPC-UA).
4. Wilma: It is in charge of guaranteeing the security of the system since it provides functions to act as a proxy within Open authorization Authentication schemes (OAuth2).
5. Perseus: This GE defines a set of rules in OCB, which makes a notification to the system or end user by means of Short Message Service (SMS), emails, or HTTP requests.

### 3.2. MPC Technique

**Figure 1:**
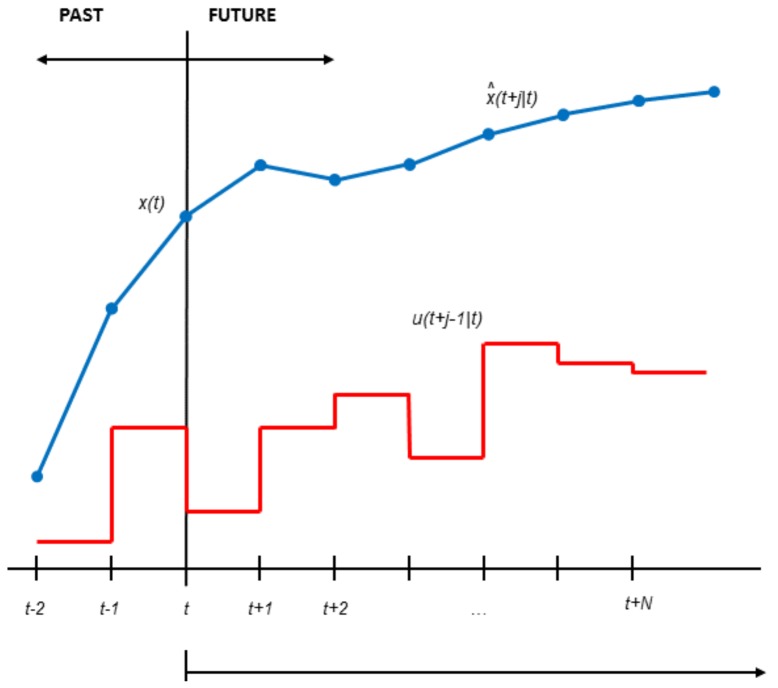
MPC strategy.

MPC is one of the most general ways of formulating a control problem. This methodology is not an explicit control technique, but rather, it involves a family of control methods based on the use of a model of the system to obtain the control signals by minimizing a given cost function [34]. The methodology can be explained according to Figure 1 and the points below:

1. The outputs of the process are predicted at each sampling time *t* along a given prediction horizon *N*, by using a model of the system. The predicted outputs, denoted by $\hat{x}\left(t+j|t\right)$ for j=1,…,N, depend on past outputs, inputs, and disturbances and on the value of future control actions u(t+j−1|t) for j=1,…,N. Note that the notation (t+j|t) is related to the predicted value of a variable at the instant time t+j, calculated with the information available at instant *t*.
2. The set of future control actions is calculated by minimizing a determined cost function.
3. The control signal u(t|t) is sent to the process while the rest of the control actions are rejected because at the next sampling time, $\hat{x}\left(t+1\right)$ will be known, allowing repeating the first step with the updated information. This methodology is known as the receding horizon concept.

It should be noted that this methodology is especially suitable for being applied in the IoT platform as it allows developing an easy control law with very limited knowledge and information of the different agents composing the distributed water network. The MPC strategy only requires the model of the system at hand, which is intrinsic controller information, and the required information to execute it for the calculation of the optimal control signals [34].

### 3.3. IoT-Architecture

**Figure 2:**
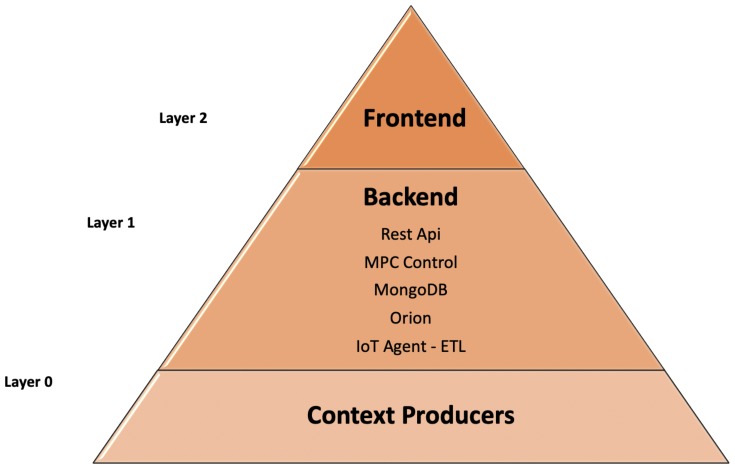
IoT platform as a pyramidal diagram.

The proposed architecture for the IoT platform can be described as a pyramidal diagram as shown in Figure 2. As can be seen, this architecture is divided into three layers that are independent of each other, allowing adding or removing services without affecting the operation of the previous layer. It is based on a back end architecture, front end, and context generators.

This architecture is defined as a cloud architecture that encompasses storage functionalities, a platform as a service, and connectivity. This kind of architecture allows the user to detach specific software or custom developments to integrate it with the system. The objective is to be able to perform services and microservices between different components of the cloud architecture. It is divided into two large back end and front end sections, which are in turn interconnected through virtual networks or the Internet. There are other parts of cloud architectures that are used, such as middleware, among other resources. The architecture can be explained according to the following points:

- Layer 0, context producers in Figure 2: IoT systems need devices that provide the data needed for ecosystem management. The IoT consists of a physical device or a network of physical devices capable of exchanging data, informing the environment to which they belong. Each device consists of an integrated microcontroller and software that can act as a sensor or actuator. The sensors are in charge of sending information about the state of certain elements available in their environment, and the actuators are in charge of carrying out actions that are directly interconnected with the data provided by the sensors. Each device is uniquely identified through the built-in computer system, but can also be identified as belonging to an existing Internet infrastructure or device network. As already mentioned, FIWARE is a system based on managing context information. The term context, applied to an intelligent Internet solution of things, is associated with the set of related elements capable of reporting the state in which the system is. Each IoT element will be represented as a unique entity within the context. IoT devices can range from simple to complex devices such as temperature, humidity, or radiation sensors or relays as actuators to launch orders.
- Layer 1, back end in Figure 2: It is the data access layer also known as the logical part of an application. It is located facing the server and is responsible for managing all services related to the data. In the back end, all the system tasks are performed, such as numerical calculations, security layer management, data access, REST services, and databases, among others. The main objective and benefit of having a back end decoupled from an application or simple architecture is the possibility of making different developments without affecting the functionality by improving the security of the system, the possibility of rescaling the service depending on the needs of the client, generating REST services allowing the back end information to interact with any client or service. In this work, the back end is formed by seven services, each independent of the others, but at the same time, forming a functional architecture. These services range from interpreters of communication protocols between sensors and the system itself, elements responsible for information management, databases, REST services, and processes responsible for process control.
- Layer 2, front end in Figure 2: This is the layer linked to the client, and therefore, it is in charge of the visualization of all the data. It is composed by a set of technologies that form the structure and design of the application. The most used programming languages for the development of this layer are JavaScript and PHP, as well as languages based on design and layout, such as HTML and CSS.

### 3.4. Case Study in Almería

**Figure 3:**
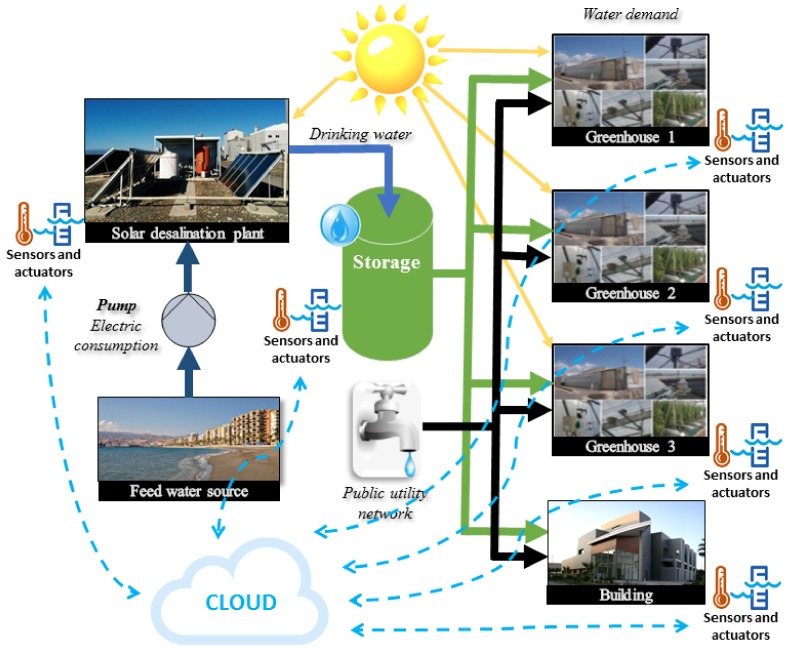
Layout of the case study.

In order to evidence the results that can be achieved with the application of the proposed platform, a case study based on three real facilities located in Almería was used. The schematic diagram of the case study is shown in Figure 3, and it was used to represent an agroindustrial district composed by several consumers agents (i.e., three greenhouses and an office building) and two water sources (i.e., a solar desalination plant and the water public utility network). Note that this small scale agroindustrial district was chosen to be representative of an industrial scale one and allow visualizing the results in a simple way. The plants included in the district are described in the following subsections.

It should be remarked that in the following subsections, the real location of the plants is established, but in real cases of application, the desalination plant should be located on the coast and the building near the greenhouses.

#### 3.4.1. Solar Desalination Plant

The solar desalination plant used as a reference in the case study was based on the Solar Membrane Distillation (SMD) facility at the Plataforma Solar de Almería (PSA, www.psa.es), in southeast Spain. A real image of the plant is included in Figure 3. This facility was fully described in [35], and it is comprised of a solar field, which is responsible for providing the thermal power required by the Membrane Distillation (MD) procedure, several MD modules, and a heat exchanger connecting both systems. The plant is totally controlled, monitoring the main variables of the distillation procedure as pressure, temperature, and flow rate. It should be commented that for the present work, it was assumed that all the control and measurement systems were based on the IoT paradigm, and they were able to receive and send information directly from or to the cloud (see Figure 3).

As stated in [10], an industrial scale MD plant (as the one used in this work) must be composed of an array of MD modules since the production of current commercial MD modules is still relatively low, around 30 L/h in optimal operating conditions. Thus, each singular MD module was connected to the array composing the overall desalination unit, as was presented in [36], so that each MD module could be turned on/off depending on the state of the valves, which allowed us to adjust the distillate production to the water demand, thus obtaining economical savings in the operation of the feed water pump. In particular, the MD module used in this work was the Aquastill one, which was totally described in [10]. As reported in that work, the module had a limited operating range in terms of temperature and feed flow rate. The temperature at the inlet of the evaporator channel of the MD module could vary between 60 and 80 ${}^{\circ }$C, whereas the flow rate between 400 and 600 L/h.

The feed solution enters though the condenser channel of the MD module, where it is preheated with the latent heat that crosses the membrane. Then, it is driven to the heat exchanger where the feed solution is heated with the fluid coming from the solar field. At last, the heated solution is flowed to the evaporator channel of the MD module, where the volatile molecules of the solution are evaporated and pass through the membrane, whereas the non-volatile ones are rejected in the form of brine. It should be remarked that in this work, it was assumed that the feed solution was water coming from the Mediterranean sea at 35 g/L (mean salinity).

For the application of the MPC technique, a model of the desalination unit is required in order to predict its total distillate production ($D_{T}$). As was presented in [10], the Aquastill module could be easily modeled by means of a polynomial equation based on empirical data. In this work, the same model was used, but it was modified by introducing binary variables ($\delta_{i}$ with $i=1,\ldots ,N_{MD}$ where $N_{MD}$ is the number of MD modules in the array fixed at 30) related to the position of the valves of each MD module *i*, assuming a value of zero when the MD module is turned off and one otherwise. In this way, the total distillate production of the overall desalination unit can be calculated as:

(1) $$\begin{array}{cc} D_{T}\left(t\right) & =\sum_{i=1}^{N_{MD}}[\left(3.24+0.072\cdot T_{cs,out}\left(t\right)-0.4896\cdot T_{feed}\left(t\right)\right)\cdot \left(1-\delta_{i}\left(t\right)\right)+(-0.024\cdot F\left(t\right)\\ & +0.0096\cdot T_{cs,out}\left(t\right)\cdot F\left(t\right))\cdot \delta_{i}\left(t\right)], \end{array}$$

where all the terms of the equation are in L/h and all the variables are presented in Appendix A. It should be noted that, when an MD module is turned on, it is operated at its maximum operating range in terms of feed flow rate (i.e., F(t)=600 L/h), which is its optimal operating point [10]. In this way, all the variables in the previous equation are constant and known, except $\delta_{i}$ with $i=1,\ldots ,N_{MD}$, which are computed by means of the MPC technique.

Moreover, as the MPC technique is aimed at reducing costs, the operating cost associated with the operation of the feed water pump must be estimated. For this aim, the electric power consumption of the feed water pump was calculated making use of the characteristic pump curve supplied by the manufacturer. The same pump as the one in [37] was used, whose characteristic curve is given by:

(2) $$P_{f}\left(t\right)=22.72\cdot c_{1}\cdot \sum_{i=1}^{N_{MD}}\left[F\left(t\right)\cdot \delta_{i}\left(t\right)\right]+39.54,$$

where $c_{1}$ is a conversion factor to transform L/h into m${}^{3}$/s so that all the terms of the equation are in kW. Note that the summation term was used to take into account all the MD modules as the total feed water flow rate was equal to the sum of feed flow rate of all the modules.

It should be remarked that this model must be executed in the cloud, integrated in the MPC strategy of the IoT architecture. For this reason, it is important to note that the information exchanged between the plant and the cloud was minimum because of the way in which the models were posed. The plant had to send information only about the temperature at the outlet of the heat exchanger for the cold side (T${}_{cs,out}$) and the feed temperature (T${}_{feed}$), and it had to receive information only about $\delta_{i}$ variables, with $i=1,\ldots ,N_{MD}$. Moreover, as the MD modules in the array were identical, the value of these variables could be given only using an integer variable (NMD) that contains the number of modules turned on at each sampling time, that is:

(3) $$NMD=\sum_{i=1}^{N_{MD}}\delta_{i}.$$

Notice also that the desalination facility was connected to a storage tank. The use of this device in this kind of systems is natural, as water is a resource that can be stored. In this way, the storage device acted as an integrator system, helping to smooth the water demands, and for this reason, its level (*L*) must also be sent to the IoT platform. This tank could be directly used or connected to the water distribution network.

#### 3.4.2. Greenhouses

The greenhouse environments included in the case study were based on the pilot greenhouse located at Experimental Station of the Cajamar Foundation (also located in southeast Spain, 40 km from the PSA), and real images of this facility can be seen in Figure 3. This pilot plant is formed by a multi-span “Parral-type” greenhouse with E-W orientation. The total surface area of the facility is 821 m${}^{2}$, among which 616 m${}^{2}$ are effective cultivation area. The cover of the greenhouse is polyethylene, and it includes an automatic ventilation system with side windows on the south and north walls. In addition, the greenhouse is equipped with a diesel aerothermal system, a biomass fueled heating system, a humidification/dehumidification system, and LED lights. The crop grows in rows with N-S-orientation, inside coconut coir bags, with three droppers and six plants each. The irrigation is performed by means of a demand tray system, which applies the irrigation to the crop periodically throughout each day. In [38], a more detailed description and explanation of the greenhouse environment can be found.

As in the desalination plant, the greenhouse was totally controlled and monitored, measuring variables such as solar irradiance, relative humidity, air temperature, CO${}_{2}$, wind direction and speed, and soil and cover temperature. For the purpose of this work, the greenhouses had to send information about their water requirements, which could be estimated by using well known models already presented in literature and based on the aforementioned measured variables, as was done in [10]. Thus, the IoT platform received information about the water needs of Greenhouses 1, 2, and 3 ($D_{GH1}$, $D_{GH2}$, $D_{GH3}$, respectively), and based on this information and according to the water production of the desalination plant ($D_{T}$) and the level of the intermediate tank (*L*), it had to decide the amount of water from the desalination plant and the public utility network used to cover the requirements of each greenhouse. Therefore, the IoT platform had to send the variables PN2GH1 and DP2GH1, PN2GH2 and DP2GH2, and PN2GH3 and DP2GH3, which were the water coming from the public utility network and the water coming from the desalination plant for Greenhouses 1, 2, and 3, respectively.

Note that for the simulations, it was assumed that the greenhouses had a tomato crop in a state of growth, with a Leaf Area Index (LAI) of 5.5 and in full production.

#### 3.4.3. Office Building

The office building incorporated in the case study was based on the Centro de Investigación de la Energía Solar (CIESOL) building (www.ciesol.es) located at the University of Almería campus, also in south east Spain, 20 km away from PSA. This building had a total surface area of 1071.91 m${}^{2}$ distributed into two floors. In addition, as in the other facilities composing the case study, CIESOL had a net of sensors to monitor the main variables affecting the building such as temperature, electricity, and water consumption. More details about the building can be found elsewhere [39].

For the management of the water network of the case study, the building had to send its water requirements ($D_{OB}$) to the IoT platform, and as happened with the greenhouses, the IoT platform had to send to the building the amount of water coming from the desalination plant (DP2OB) and the public utility network (PN2OB).

### 3.5. Application of the IoT Platform to the Case Study

**Figure 4:**
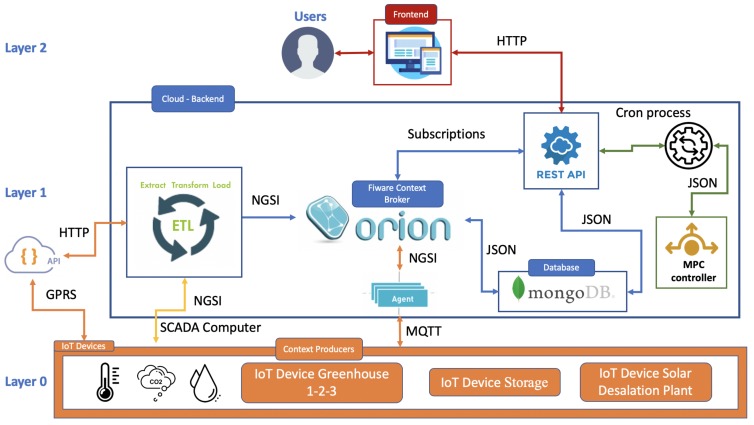
Architecture of the IoT platform.

The architecture proposed in this paper for the management of the agroindustrial district presented above in terms of water is shown in Figure 4. As mentioned in Section 3.3, it was divided into three layers: context producers, back end, and front end. It is important to remark that the proposed architecture was based on that already developed for the IoF2020UC4.2 Vegetables project [40,41], with the particularity of applying control techniques, among other data extraction functionalities adapted to this system.

#### 3.5.1. Layer 0 Context Producers

All the devices and elements in charge of generating context information were available in this layer. Different kind of actuators and sensors were available in each plant forming the agroindustrial district. These sensors were connected to different commercial IoT stations or data acquisition systems. There were six types of context information production scenarios distributed in different parts of the proposed scenario: a solar desalination plant, three Parral-type greenhouses, an office building, and a water storage device. These facilities sent the measurements collected by the sensors to the cloud through the MQTT or HTTP requests, depending on the data extraction format. MQTT is one of the most extended communication protocols used in the IoT paradigm due to its lightness and simplicity, which are mainly produced by the power limitations in the devices and the bandwidth. It is based on the Transmission Control Protocol/Internet Protocol (TCP/IP) protocol, which reuses already open connections, unlike the HTTP 1.0 protocol, which makes new connections. Its operation is based on push messaging as editor/subscriber and themes. In addition, it has a central broker that manages the registration of the client’s connections, allowing subscriptions to different topics. On the one hand, MQTT will be used for sending smart sensors to OCB; on the other hand, HTTP will be used for sending data from the acquisition systems to OCB.

#### 3.5.2. Layer 1 Backend

This section describes the layer in charge of managing all the operations of the IoT system. Each of the services that make up this data layer was independent of the others, thus allowing updates and developments without affecting the performance and stability of the system. It was formed by a set of services that interacted with each other to form the ecosystem based on FIWARE:

- Extract context information: This service is responsible for extracting, transforming, and sending the data to the IoT system. There are two services calledIDAS (represented by the block agent in Figure 4) and Extract, Transform, and Load (ETL), responsible for translating the information that comes from the sensors to the NGSI standard (see Figure 4). The objective of having two interpretation systems is to provide a solution that is as complete as possible. The context information can come from three data sources: commercial systems with their own REST services (represented in the form of a cloud API in Figure 4 based on General Packet Radio Service (GPRS) communication), data acquisition systems connected to a PC, and smart sensors prepared to send these data to the cloud. Commercial stations are available from the following manufacturers: Hortisys of the Hispatec model, iMetos of the Pessl model, iMetos ECO D3, and the Hops model. The first service called ETL (see Figure 4) is responsible for extracting data from REST services of commercial stations or data acquisition systems connected to a PC, making the transformation to the NGSI standard, and then, sending it directly to OCB. The second service called IDAS (represented by the block agent in Figure 4) is an enabler developed by FIWARE, which comes into use in the case of having intelligent sensors sending data directly to OCB, performing the function of interpreting the MQTT communication protocol that these sensors use for the FIWARE NGSI standard.
- OCB: It is the core of the architecture (see Figure 4), and it is tasked with handling the context information. It acts as an intermediary between producers (publishers) and consumers (subscribers). It is based on the NGSI specification defined by OMA. The data model is based on entities, attributes, and metadata. There are six entities for each of the context producers: solar desalination plant, water storage tank, Greenhouse 1, Greenhouse 2, Greenhouse 3, and finally, office building. Table 1 shows exclusively each of the entities with their respective attributes and metadata necessary to perform the MPC technique. In addition, each of these entities has a set of sensors and actuators, sending the information to OCB. In Table 1, the internal attributes can be seen of the variables generated by these entities with the needs of the system, while the controller external attributes column includes those variables generated when applying the MPC techniques (each of these variables are explained in the Section 3.4). The objective of including the controller external attributes within the entity is to be able to link the system actuator with this attribute, allowing the action to be performed directly. This fact creates a new entity MPC controller. To differentiate between different types of stations in the same entity, FIWARE-Service and FIWARE-ServicePathare used, permitting hierarchical scopes in the same entity. The first element defines the name of the plant, whereas the second one, the name of the station or set of sensors. Each one of the sensors is detailed independently with its name and value in the attributes. Within these attributes, metadata are available, giving the possibility of creating fields such as the date when reading data and the common name, among others.
- REST API services: There are two types of services, one in charge of carrying out the persistence of the data and the other that manages the requests on behalf of the client. These two services are encapsulated within the REST API module (see Figure 4). As mentioned above, OCB allows entities to subscribe, and this is because the first REST service carries out a series of subscriptions to each of the available entities. When one of these entities undergoes a change in any of its attributes, this will be notified to all the services subscribed to the entity. An HTTP request will be sent to the persistence service, which will check if the data and date already exist in the system before saving the measurement. The second service offers a REST API, which is exposed to the end user through an application. The end user will make the necessary requests to the system, and it will return the requested response in JSON format.
- Cron process: This service is a Unix cron process (see Figure 4) designed to run periodically every 15 min. Its objective is to retrieve the data from the previous REST service in charge of obtaining the database information and send it to the controller to perform the MPC control technique. Once the controller finishes the execution, this same service sends the parameters of Table 1’s controller external attributes column to its corresponding entity, updating the OCB information and thus the actuator with the new execution parameters. When there is a subscription to each entity, it notifies again a change to the REST service in charge of persisting the information and performs the action of saving in the database.
- MPC controller: This is the service in charge of carrying out the optimal management of the water resources (see Section 3.4). This service (see Figure 4) is controlled by a cron process and allows consulting the needs of each system through the REST service and inserting the new instructions in the control column of the external attributes of each entity (see Table 1). All the details about the implementation of the MPC controller are presented in Appendix B.
- Database: This architecture is supported by a non-relational database. It is based on MongoDB (see Figure 4), which allows managing a large volume of data, easy scalability, and a dynamic data model. The latter is essential in this architecture, as it allows adding or removing sensors from each entity without the risk of affecting the operation of the system.

#### 3.5.3. Layer 2 Front End

This is the data visualization layer. The user makes the necessary requests to the system from this service. The objective of separating the front end as an independent service is to give versatility to the system of creating a web, desktop, or hybrid application. Any changes made to the application do not affect the operation of the IoT system.

## 4. Results and Discussion

### 4.1. Simulation Results

To evidence the results that could be attained with the application of the designed IoT platform in an agroindustrial district, a simulation was carried out in MATLAB 2018b with the YALMIP toolbox. To perform the simulation, real Meteorological data from PSA and from Experimental Station of Cajamar Foundation on the day 20 July 2017 were used. The models of the desalination plant and the greenhouses presented in [37,38] respectively were simulated with the actual meteorological data to obtain $T_{cs,out}$ and $D_{GH1}$ and $D_{GH2}$ and $D_{GH3}$. Moreover, in order to add difficulty to the management problem, different sizes were used for simulating the greenhouses, so that the size of Greenhouses 1 and 2 was fixed at 1 ha, whereas that of Greenhouse 3 at 0.5 ha. Besides, the irradiance data for Greenhouse 3 were shifted forward 15 min in the simulations. Conversely, real water consumption data of the aforementioned day from CIESOL building were used for $D_{OB}$. These data were also scaled considering a building with a surface area of 400 m${}^{2}$. Notice that to test the proposed strategy, two days were simulated duplicating the data, but augmenting the irradiance profiles used for simulating Greenhouses 2 and 3 in the second day in order to augment their water demand.

The configuration parameters of the MPC controller were set as: (i) sampling time (Ts) fixed at 15 min, which was chosen taking into account the dynamics of the office building, desalination, and greenhouses, and (ii) prediction horizon (*N*) fixed at six. Note that the prediction horizon must be selected large enough in order to catch the process transients. However, it should be taken into account that as the prediction horizon (*N*) increases, so does the amount of decision variables included in the optimization problem and, therefore, the computational time. For this reason, the prediction horizon was chosen considering a tradeoff between these two issues and after exhaustive simulations.

**Figure 5:**
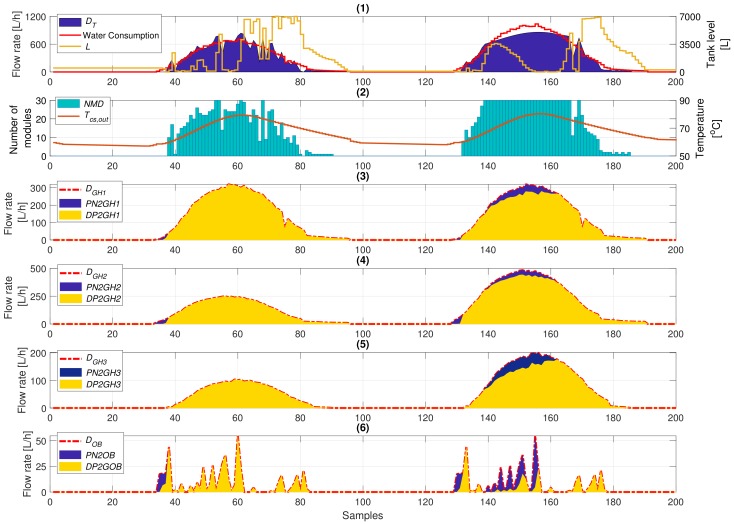
Simulation results. All the variables are according to Appendix A.

The results obtained from the simulations are presented in Figure 5. It is worth noting that the costs related to the water coming from the public utility network are higher than those related to the operation of the feed pump of the desalination plant, even when all the MD modules are in operation. Therefore, the optimal management consists of feeding the consumer agents by the desalination plant whenever possible. In this way, at the end of the day, the storage tank level must be zero or very close to this value. Consequently, the initial state of the storage tank level was set to 50 L (see Figure 5(1)).

In this way, around Sample 34, the office building started demanding water (see Figure 5(6)), and in Sample 35, also Greenhouses 1 and 2 (see Figure 5(3),(4)). Nevertheless, as the temperature $T_{cs,out}$ was below $T^{*}$ at that moment (see Figure 5(2)), the desalination plant could not still produce fresh water, and the water requirements were met by the remaining water in the tank and water coming from the water utility public network. At Sample 37, the temperature $T_{cs,out}$ was above $T^{*}$, and the desalination plant was turned on by the MPC controller (see Figure 5(2)). For the rest of the operation on this day, the greenhouse water necessities and the ones of the office building were satisfied by the desalination plant. The benefit of using an MPC controller was especially shown at the end of the day. In that moment, and before $T_{cs,out}$ reaching $T^{*}$, the MPC controller increased the amount of modules turned on (see Sample 70 in Figure 5(2)) trying to augment the water stored in the tank, thus avoiding the use of water coming from the public utility network when all the MD modules were turned off.

In the second operating day, the beginning of the operation was similar to that from the previous day, but on this occasion, there was no water left in the storage tank, so that only water coming from the public utility network was used to fulfil the water needs. Moreover, as shown in Figure 5(3),(4), the water necessities of Greenhouses 2 and 3 were higher than in the first operating day. This fact caused the MPC controller to turn on all the MD modules included in the array (see Figure 5(2)). However, the water production of the desalination facility was not enough to fulfil the water needs, and for this reason, the water production was complemented with water coming from the public utility network, as shown in Figure 5(3)–(6). Finally, at the end of the day, the same fact happened as in the first day: the water level of the tank was increased to meet the water needs without using the public utility network when the desalination plant was turned off.

### 4.2. Comparative Operating Cost Analysis

To illustrate the benefits achieved in terms of operating costs with the application of the proposed IoT platform in real agroindustrial districts, the results shown in the previous subsection were compared to those obtained using a manual operation. The manual operation consisted of turning on all the MD modules as long as $T_{cs,out}$ was higher than $T^{*}$, which was not optimal from the point of view of the operation of the desalination plant, as it was producing more water than necessary and, therefore, consuming more electricity (increasing costs). The results are presented in Table 2.

As can be seen, the economic savings were considerable. The costs associated with the operation of the desalination facility were reduced by 87%, whereas the total costs by 75% with the use of the proposed method. This was directly reflected in the specific cost of the water demanded, as it can be seen in Table 2 how with the application of the proposed method, the cost per unit of water demanded was 0.44 €/m${}^{3}$, whereas by using a non-optimal manual procedure, the cost was 1.51 €/m${}^{3}$.

## 5. Conclusions

This work addressed the development of an IoT based water management architecture to be applied in agroindustrial districts including desalination plants, connection to the public utility network, and several consumer agents. The core of the platform was based on the use of FIWARE and an MPC controller that reflected the operational strategy in real time. Simulation tests using a case study based on three real facilities located in Almería were performed. The obtained results allowed us to draw the following conclusions:

1. The use of enabling technologies such as IoT on agroindustrial districts could be an effective tool to carry out the optimal management of the heterogeneous resources required by the elements that make up these environments.
2. In particular, the application of the proposed method to the case study demonstrated how an optimal management of the water resources could be done, adapting the water production of the desalination plant to the water demand of the consumer agents and minimizing the use of water coming from the public utility network, thus making a proper use of desalination facilities.
3. The comparative cost analysis performed with a manual operation showed how around 75% of the operational cost could be saved. In this way, the cost per unit of water demanded in the agroindustrial district was reduced with the application of the proposed strategy from 1.51 €/m${}^{3}$ (cost of the manual operation) to 0.44 €/m${}^{3}$. This could be very relevant to maintain the economic sustainability of the agricultural system of Almería.

In relation to future work, the speed of computing and latency when replicating the control system on edge computing versus the current cloud computing could be compared. In this way, it would be possible to detect which system performs better control and what the response times would be. Furthermore, it would be interesting to carry out an economic viability analysis taking into account the different IoT systems, transport infrastructures, and desalination costs.

## Figures and Tables

**Table 1:** Entities of OCB FIWARE required for MPC control.

Entities	Internal Attributes	Controller External Attributes	Metadata
Solar desalination plant	T${}_{cs,out}$, T${}_{feed}$	NMD	Common name, Unix date
Water storage tank	*L*	-	Common name, Unix date
Greenhouse 1	$D_{GH1}$	PN2GH1, DP2GH1	Common name, Unix date
Greenhouse 2	$D_{GH2}$	PN2GH2, DP2GH2	Common name, Unix date
Greenhouse 3	$D_{GH3}$	PN2GH3, DP2GH3	Common name, Unix date
Office Building	$D_{OB}$	PN2OB,DP2OB	Common name, Unix date

**Table 2:** Comparative operating cost results. TPNCis the total operating cost associated to the water coming from the public utility network; TDPCis the total operating cost of the desalination facility; TC is the Total Cost; and SC is the Specific Cost, that is the cost per unit of water demanded in the agroindustrial district.

Management Method	TPNC (€)	TDPC (€)	TC (€)	SC (€/m${}^{3}$)
IoT platform	2.60	3.01	5.61	0.44
Manual	18.70	0.20	18.90	1.51

## Abbreviations

The following abbreviations are used in this manuscript:

CIESOL	Centro de Investigación de la Energía Solar
CoAP	Constrained Application Protocol
CHROMAE	Control and Optimal Management of Heterogeneous Resources in Agroindustrial production
	districts integrating renewable Energies
ETL	Extract, Transform, and Load
GEs	Generic Enablers
GPRS	General Packet Radio Service
HTTP	Hypertext Transfer Protocol
ICT	Information and Communication Technology
IDAS	Intelligence Data Advanced Solution
IoT	Internet of Things
JSON	JavaScript Object Notation
LAI	Leaf Area Index
LWM2M	LightweightM2M
MD	Membrane Distillation
MPC	Model Predictive Control
MQTT	Message Queue Telemetry Transport
NGSI	Next Generation Services Interface
OAuth2	Open Authorization
OCB	Orion Context Broker
OPC-UA	Object Linking and Embedding for Process Control-Unified Architecture
OMA	Open Mobile Alliance
PA	Precision Agriculture
PSA	Plataforma Solar de Almería
SMS	Short Message Service
TCP/IP	Transmission Control Protocol/Internet Protocol

## Appendix A

### Nomenclature

**Variable**	**Description**	**Value and Units**
$c_{1}$	Conversion factor to transform L/h into m${}^{3}$/s	2.7$\cdot 10^{-7}$ m${}^{3}\cdot$ h/(L·s)
$c_{2}$	Conversion factor to transform min into h	0.016 h/min
$c_{3}$	Conversion factor to transform L/h into m${}^{3}$/min	1.63$\cdot 10^{-5}$ m${}^{3}\cdot$ h/(L·min)
$C_{PN}$	Total cost associated to the operation of the feed water pump of the desalination facility	€
$C_{PN}$	Total cost of the water coming from the public utility network	€
$D_{T}$	Total distillate production of the desalination plant	L/h
$D_{GH1}$	Water demand of Greenhouse 1	L/h
$D_{GH2}$	Water demand of Greenhouse 2	L/h
$D_{GH3}$	Water demand of Greenhouse 3	L/h
$D_{OB}$	Water demand of the office building	L/h
DP2GH1	Water coming from the desalination plant to Greenhouse 1	L/h
DP2GH2	Water coming from the desalination plant to Greenhouse 2	L/h
DP2GH3	Water coming from the desalination plant to Greenhouse 3	L/h
DP2OB	Water coming from the desalination plant to the office building	L/h
$E_{p}$	Electricity price	0.14€/kWh
*F*	Feed flow rate	L/h
*L*	Water level of the storage tank	L
$L_{min}$	Minimum water level of the storage tank	L
$L_{max}$	Maximum water level of the storage tank	L
$N_{MD}$	Number of MD modules in the desalination unit	30
*m*	Constant 1 used in the MPC problem	−1000
*M*	Constant 2 used in the MPC problem	1000
NMD	Integer variable containing the number of MD modules turned on at each sampling time	-
$P_{f}$	Power consumption of the feed water pump of the desalination plant	kW
PN2GH1	Water coming from the public utility network to Greenhouse 1	L/h
PN2GH2	Water coming from the public utility network to Greenhouse 2	L/h
PN2GH3	Water coming from the public utility network to Greenhouse 3	L/h
PN2OB	Water coming from the public utility network to the office building	L/h
$T_{cs,out}$	Temperature at the outlet of the heat exchanger, cold side	°C
$T_{feed}$	Feed water temperature	°C
$T_{s}$	Sampling time	15 min
$T^{*}$	Minimum temperature required to operate the desalination plant	60 °C
$W_{p}$	Price of water coming from the public utility network	0.50€/kWh
$\beta_{i}$	Auxiliary binary variable used in the MPC problem related to the valve position of each MD module *i*	0–1
$\delta_{i}$	Valve position of each MD module *i*	0–1
γ	Auxiliary binary variable used in the MPC problem related to the operational constraints	0–1

## Appendix B

### MPC Control Formulation

The MPC control problem to be solved at each sampling time can be posed as a Mixed Integer Linear Programming (MILP) optimization problem as follows:

(A1) $$\min\;J={\sum_{j=1}}^{N}\left[{\hat{C}}_{\mathrm{PN}}\left(t+j|t\right)+{\hat{C}}_{\mathrm{DP}}\left(t+j|t\right)\right],$$

subjected to ∀j=1,…,N:

(A2) $${\hat{D}}_{T}\left(t+j|t\right)=\sum^{N}\mathrm{MDi}=1[\left(3.24+0.072\cdot {\hat{T}}_{cs,out}\left(t+j|t\right)-0.4869\cdot {\hat{T}}_{\mathrm{feed}}\left(t+j|t\right)\right)\cdot \left(1-\beta_{i}\left(t+j-1|t\right)\right)\;+(-0.024\cdot F\left(t+j-1|t\right)+0.0096\cdot {\hat{T}}_{cs,out}\left(t+j|t\right)\cdot F\left(t+j-1|t\right))\cdot \beta_{i}\left(t+j-1|t\right)],$$

(A3) $$\beta_{i}\left(t+j-1|t\right)\in \left\{0,1\right\},\;\;\forall i=1,\ldots ,N_{MD},$$

(A4) $$-m\cdot \gamma \left(t+j-1|t\right)\leq ({\hat{T}}_{cs,out}\left(t+j|t\right)-T^{*})-m,$$

(A5) $$-M\cdot \gamma \left(t+j-1|t\right)\leq -({\hat{T}}_{cs,out}\left(t+j|t\right)-T^{*}),$$

(A6) γ(t+j−1|t)∈{0,1},

(A7) $$-\beta_{i}\left(t+j-1|t\right)+\delta_{i}\left(t+j-1|t\right)\leq 0,\;\;\forall i=1,\ldots ,N_{MD},$$

(A8) $$-\gamma \left(t+j-1|t\right)+\delta_{i}\left(t+j-1|t\right)\leq 0,\;\;\forall i=1,\ldots ,N_{MD},$$

(A9) $$\beta_{i}\left(t+j-1|t\right)+\gamma \left(t+j-1|t\right)-\delta_{i}\left(t+j-1|t\right)\leq 1,\;\;\forall i=1,\ldots ,N_{MD},$$

(A10) $$\delta_{i}\left(t+j-1|t\right)\in \left\{0,1\right\},\forall i=1,\ldots ,N_{MD},$$

(A11) $$\begin{array}{c} \hat{L}\left(t+j|t\right)=\hat{L}\left(t+j-1|t\right)+c_{2}\cdot T_{s}\cdot [{\hat{D}}_{T}\left(t+j|t\right)-\hat{DP2GH1}\left(t+j|t\right)-\hat{DP2GH2}\left(t+j|t\right)\\ -\hat{DP2GH3}\left(t+j|t\right)-\hat{DP2OB}\left(t+j|t\right)], \end{array}$$

(A12) $$L_{min}\leq \hat{L}\left(t+j|t\right)\leq L_{max},$$

(A13) $${\hat{D}}_{GH1}\left(t+j|t\right)=\hat{DP2GH1}\left(t+j|t\right)+\hat{PN2GH1}\left(t+j|t\right),$$

(A14) $${\hat{D}}_{GH2}\left(t+j|t\right)=\hat{DP2GH2}\left(t+j|t\right)+\hat{PN2GH2}\left(t+j|t\right),$$

(A15) $${\hat{D}}_{GH3}\left(t+j|t\right)=\hat{DP2GH3}\left(t+j|t\right)+\hat{PN2GH3}\left(t+j|t\right),$$

(A16) $${\hat{D}}_{OB}\left(t+j|t\right)=\hat{DP2GOB}\left(t+j|t\right)+\hat{PN2GOB}\left(t+j|t\right),$$

(A17) $${\hat{C}}_{DP}\left(t+j|t\right)=\left(22.72\cdot c_{1}\cdot \sum_{i=1}^{N_{MD}}\left[F\left(t+j-1|t\right)\cdot \delta_{i}\left(t+j-1|t\right)\right]+39.54\right)\cdot T_{s}\cdot c_{2}\cdot E_{p},$$

(A18) $$\begin{array}{c} {\hat{C}}_{PN}\left(t+j|t\right)=(\hat{PN2GH1}\left(t+j|t\right)+\hat{PN2GH2}\left(t+j|t\right)+\hat{PN2GH3}\left(t+j|t\right)\\ +\hat{PN2GOB}\left(t+j|t\right))\cdot c_{3}\cdot T_{s}\cdot W_{p}. \end{array}$$

In this formulation, the objective function (Equation (A1)) aims to reduce the operational costs of the agroindustrial district in terms of water, considering the cost of the water coming from the public utility network ($C_{PN}$) and that of the feed water pump of the desalination plant ($C_{DP}$). Note that other types of costs in the desalination plant, as the ones of the solar field feeding it, have not been taken into account as they were already optimized in the previous published work [37]. The optimization problem is subjected to a set of process and operational constraints, which are presented in Equations (A2)–(A18).

Firstly, the set of constraints in Equations (A2)–(A10) is related to the desalination plant. In this set, the constraints in Equations (A2) and (A3) are used to compute the distillate production ($\hat{D_{T}}\left(t+j|t\right)$) along the prediction horizon. Note that the model presented in Equation (1) is employed, but an auxiliary binary variable $\beta_{i}\left(t+j-1|t\right)$ with $i=1,\ldots ,N_{MD}$ for taking into account the MD modules states (turned on/off) is used instead of the actual variable $\delta_{i}\left(t+j-1|t\right)$ with $i=1,\ldots ,N_{MD}$. This is because the modules can be only turned on if the temperature required to operate them ($T^{*}$= 60 °C) is reached. Thus, this statement is included in the optimization problem using the set of constraints in Equations (A4)–(A6), so that another auxiliary binary variable γ(t+j−1|t) takes the value of one if the aforementioned statement is true and zero otherwise. Finally, the value of the actual variable related to the valve position of each MD module ($\delta_{i}\left(t+j-1|t\right)$ with $i=1,\ldots ,N_{MD}$) is computed at each instant time as $\delta_{i}\left(t+j-1|t\right)=\beta_{i}\left(t+j-1|t\right)\cdot \gamma_{i}\left(t+j-1|t\right)\;\forall i=1,\ldots ,N_{MD}$. This multiplication is formulated in the problem by means of a set of linear constraints (see Equations (A7)–(A10)).

Secondly, the constraints in Equations (A11) and (A12) are related to the tank level. The first one is used to compute the level ($\hat{L}\left(t+j|t\right)$) along the prediction horizon, which is calculated based on the level of the previous instant time ($\hat{L}\left(t+j-1|t\right)$), the water that is received from the desalination plant ($\hat{D_{T}}\left(t+j|t\right)$), and the water that is sent to to the office building and Greenhouses 1, 2, and 3 ($\hat{DP2OB}\left(t+j|t\right)$, $\hat{DP2GH1}\left(t+j|t\right)$, $\hat{DP2GH2}\left(t+j|t\right)$, and $\hat{DP2GH3}\left(t+j|t\right)$, respectively). The second one defines the maximum and minimum level of the tank.

Thirdly, the constraints in Equations (A13)–(A16) define the relationship between producers and consumer agents, so that the water demanded by each consumer agent must be covered by the sum of the water that it receives from the desalination plant and from the water public utility network.

Fourthly, the constraints in Equations (A17) and (A18) are used to calculate the cost related to the operation of the feed water pump of the desalination plant ($C_{DP}$) and that of the water coming from the public utility network ($C_{PN}$) along the prediction horizon.

Once the overall MPC problem is formulated, it should be pointed out that the decision variables of the optimization problem are $\delta_{i}$ and $\beta_{i}\;\forall i=1,\ldots ,N_{MD}$, γ, DP2GH1, PN2GH1, DP2GH2, PN2GH2, DP2GH3, PN2GH3, DP2OB, and PN2OB. Conversely, *L*, $T_{cs,out}$, $T_{feed}$, $D_{GH1}$, $D_{GH2}$, $D_{GH3}$, and $D_{OB}$ are state variables whose values change according to the operational conditions of the desalination plant and the greenhouses and office building, respectively. In the simulations, real data were used for predicting these variables.
